# Editorial: Recent advances and new challenges in minimally invasive surgery and chemotherapy for colorectal cancer

**DOI:** 10.3389/fsurg.2023.1341194

**Published:** 2024-01-05

**Authors:** Hiroki Hashida

**Affiliations:** Department of Surgery, Kobe City Medical Center General Hospital, Kobe, Japan

**Keywords:** colorectal cancer, chemotherapy, minimally invasive surgery, laparoscopic surgery, robotic surgery

**Editorial on the Research Topic** Recent advances and new challenges in minimally invasive surgery and chemotherapy for colorectal cancer

## Introduction

The landscape of colorectal cancer treatment has been reshaped significantly in recent years, primarily driven by advancements in minimally invasive surgery (MIS) and a deeper understanding of cancer biology. The Research Topic “Recent Advances and New Challenges in Minimally Invasive Surgery and Chemotherapy for Colorectal Cancer” in Frontier in Surgery offers a panoramic view of the state-of-the-art practices and emerging challenges in this field. This editorial aims to synthesize the key findings from the six pivotal articles that constitute this collection, providing insights into the ongoing evolution of colorectal cancer management.

## Prophylactic ileostomy sites in laparoscopic rectal cancer surgery

The first article addresses a critical question in laparoscopic surgery for rectal cancer: the optimal site for prophylactic ileostomy. Through a systematic review and meta-analysis, this study evaluates whether the ileostomy should be created at the specimen extraction site or a new site. The findings have significant implications for postoperative recovery and complication rates, providing valuable guidance for surgeons (Zheng et al.).

## Minimally invasive surgery and incisional surgical site infections

The second article delves into the capacity of MIS to reduce the occurrence of incisional surgical site infections in colorectal cancer and other gastroenterological malignancies. This exploration is crucial, given that surgical site infections remain a leading cause of morbidity in surgical oncology. The study provides robust evidence supporting the adoption of MIS techniques in colorectal cancer surgeries (Yamamoto et al.).

## Advances in diagnosing lymph node metastasis in rectal cancer

The third article presents a comprehensive review of the progress in diagnosing lymph node metastasis in rectal cancer, a pivotal factor in staging and treatment planning. The review highlights the latest diagnostic tools and techniques, underscoring their role in improving the accuracy of lymph node assessment (Peng et al.).

## Prolapsing technique and one-stitch ileostomy

The fourth article offers a retrospective study on the integration of the prolapsing technique and a one-stitch method for ileostomy during laparoscopic low anterior resection for rectal cancer. This innovative approach could significantly impact the ease of ileostomy closure and patient comfort post-surgery (Li et al.).

## Two-port laparoscopic surgery in sigmoid and upper rectal cancer

The fifth article explores the clinical application of two-port laparoscopic surgery for resection in sigmoid colon and upper rectal cancer. This minimally invasive approach could represent a significant advancement in surgical technique, offering a less invasive option with potential benefits in patient recovery and cosmetic outcomes (Jiang et al.).

## Preoperative systemic immune inflammatory index in colorectal cancer

Finally, the sixth article focuses on the prognostic evaluation of the preoperative systemic immune inflammatory index in patients with colorectal cancer. This study highlights the growing importance of systemic inflammatory markers as prognostic tools, potentially aiding in treatment stratification and personalized patient management (Zhang et al.).

Regrettably, despite the robust discussions and insights offered throughout the articles, a dedicated discourse on robotic surgery's role and future was not fulfilled within this collection. It remains a pivotal aspect of minimally invasive surgery that warrants further exploration. We look forward to future submissions that can fill this gap, offering new perspectives and studies which can be found here.

In conclusion, this Research Topic presents a rich tapestry of current advances and challenges in the field of colorectal cancer treatment, particularly in the realms of minimally invasive surgery and chemotherapy. Each article contributes a unique perspective, collectively enhancing our understanding of this complex and evolving landscape. As we continue to push the boundaries of what is possible in colorectal cancer care, these studies serve as a beacon, guiding us towards more effective, less invasive, and highly personalized treatment modalities.

I express my gratitude to all contributors who have enriched this theme with their expertise and insights. Finally, I would like to thank co-editor, Professor Nobu Oshima.

On behalf of the Topic Editors,

Hiroki Hashida

